# Juvenile Hormone Titer Versus Juvenile Hormone Synthesis in Female Nymphs and Adults of the German Cockroach, *Blattella germanica*

**DOI:** 10.1673/031.006.4301

**Published:** 2006-11-27

**Authors:** Karl Treiblmayr, Nuria Pascual, Maria-Dolors Piulachs, Thomas Keller, Xavier Belles

**Affiliations:** ^1^ University of Salzburg, Department of Organismic Biology, Hellbrunnerstrasse 34, A-5020 Salzburg, Austria.; ^2^ Department of Physiology and Molecular Biodiversity, Institut de Biologia Molecular de Barcelona (CSIC), Jordi Girona 18–26, 08034 Barcelona, Spain.; ^3^ University of Salzburg, Institute of Forensic Medicine, Ignaz Harrerstr, 79, 5020 Salzburg, Austria

**Keywords:** hemolymph juvenile hormone titer, CA corpora allata, JH juvenile hormone

## Abstract

Patterns of juvenile hormone have been intensively studied in the cockroach *Blattella germanica* under different physiological situations. However, data have been mainly obtained *in vitro*, and refer to hormone synthesized by isolated *corpora allata*, whereas information available on hormone concentration in the hemolymph is restricted to adult females. In order to complement our studies *in vitro*, we have measured juvenile hormone titer in the hemolymph of *B. germanica* females in four characteristic physiological situations: penultimate and last instar nymphs, adults during the first vitellogenic cycle, and adults transporting egg cases (ootheca). In general, a significant positive correlation between rates of hormone synthesis and concentration in the hemolymph is observed. The main disparities appear in the penultimate day of the period of ootheca transport, where titer is high whereas synthesis is low, and on day 6 of the first vitellogenic cycle, where synthesis increases whereas titer decreases. At these stages, the observed disparities between synthesis and titer might be explained by differential action of degradation enzymes.

## Introduction

Changes in juvenile hormone (JH) titer during development modulate metamorphosis in both hemimetabolous and holometabolous insects, whereas in adult females, high JH titer is associated with vitellogenesis and oocyte maturation in most species (Bellés 2004; Raikhel et al. 2005). In addition, in cockroaches such as *Blattella germanica*, that transport an egg case, the ootheca, externally until egg hatching, JH production is very low during the period of transport, which reduces oocyte growth thus avoiding the premature deposition of the ootheca (Osorio et al. 1997).

The unusual reproductive physiology and oviposition behavior of *B. germanica* has led to study the mechanisms of JH regulation, and a considerable amount of information on this topic is available. Most of the data concern JH rates of biosynthesis by the *corpora allata* (CA), either in the adult during the first vitellogenic cycle (Bellés et al. 1987; Gadot and Schal 1989; Bellés et al. 1994; Maestro et al. 1994), as well as during the two last nymphal instars (Cruz et al. 2003). Conversely, much less information is available on JH concentration in the hemolymph. The first study identified JH III as the JH homolog present in the adults of *B. germanica*, and also reported preliminary results on JH titer in the hemolymph during the first reproductive cycle of adult females (Camps et al. 1987). Subsequent measurements *in vivo* were carried out on adult females during the first reproductive cycle and during the period of ootheca transport (Sevala et al. 1999). Data on hemolymph JH titer in nymphs *of B. germanica* are lacking.

Given that hemolymph levels of JH is what really counts when considering biological effects, we have measured JH titer in the hemolymph of *B. germanica* females in four physiological situations related to metamorphosis and reproduction: penultimate and last instar nymphs, adults during the first vitellogenic cycle, and adults transporting the ootheca. Data obtained will complement our previous results that described JH synthesis by the CA, and will help to better understand the effects of this hormone in our model species.

## Materials and Methods

### Insects

Specimens of *B. germanica* were obtained from a colony reared in the dark at 30 ± 1 °C and 60–70% RH. Freshly ecdysed nymphs or adult females were selected and used at the appropriate ages. Female nymphs were sexed and staged as previously described (Ross and Cochran 1960). All dissections and tissue sampling were carried out on carbon dioxide-anaesthetized specimens.

### Hemolymph sampling

Hemolymph was collected from each specimen, from penultimate nymphal instar to adult stage, with a calibrated micropipette applied to a cut femur. Hemolymph from 2 to 8 individuals of each age was pooled, and 2 to 6 pools were analyzed.

### Identification and quantification of hemolymph juvenile hormone

Hemolymph samples (5–10 µl) were diluted in 2 ml methanol containing 0.5 pmol ethyl ester homologues of JH I and JH III as internal standards. JH measurement was performed as previously reported (Rembold and Lackner 1985). Essentially, the method is based on extraction in iso-octane, adsorption-desorption sequences involving derivatization of JHs and measurement by combined gas chromatography-selected ion monitoring mass spectrometry (GC-MS-SIM). The method measures free and bound JH, and is related to that of Bergot et al. (1981), although it follows a different system of derivatization of JH. Before processing the hemolymph, 0.5 or 5.0 pmol of JH I and JH III ethyl standards were added to each sample, depending, respectively, on whether JH production by CA was high (penultimate nymphal instar and transition to last nymphal instar, adult female in the first vitellogenic cycle, transition from the period of ootheca transport to the second vitellogenic cycle) or low (last nymphal instar, adult female in the period of ootheca transport).

A first series of measurements were carried out with a Carlo Erba HRCG/MS gas chromatograph coupled on-line with a Varian (www.varianinc.com) CH-7 A-double focused sector field mass spectrometer. A falling needle injector was used to inject 5 µl of sample. The injector temperature was 300deg;C, the initial oven temperature was 200°C, and oven temperature program moved from 200°C to final 290°C (11°C/min). The mass measurement was started 5 min after sample injection, and the different JHs were eluted within the next 3 min. A second series of measurements were carried out with an Agilent (www.agilent.com) GC 6890 N gas chromatograph coupled with an Agilent MSD 5973 mass detector. A splitless injection mode was used to inject 2 µl of sample. An Agilent 7683 autosampler was used for injection into the gas chromatograph. In this case the injection port temperature was 300°C, the initial oven temperature was 100°C, and oven temperature program moved from 100°C to final 300°C (30°C/min). The different JHs were eluted within the next 3 min. In both series of measurements, a fused silica capillary column (J&W DB 1, film thickness 0.25µm, length 30 m, 0.25 µm ID) was used to separate the JH derivatives, and the limit of detection was 0.02 pmol of JH. Both series of measurements gave similar results.

### Incubation of corpora allata and quantification of juvenile hormone synthesis

Individual *corpora cardiaca-corpora allata* complexes from females of known ages, were incubated in 100 µl of TC199 medium (Sigma, www.sigmaaldrich.com), containing L-methionine (0.1 mM), Hank's salts, Hepes (20 mM) plus Ficoll (20 mg/ml), to which L-[3H-methyl]-methionine (Amersham, www.apbiotech.com) had been added to achieve a final specific activity of 7.4 Gbq/mmol. JH III, which is the native JH in the adult female of *B. germanica* (Camps et al. 1987), was quantified after 3 h of incubation. At the end of the incubation, JH III synthesis was determined by organic solvent extraction of the medium plus the incubated glands, followed by JH purification by thin layer chromatography, as previously described (Piulachs and Couillaud 1992).

## Results

### JH in female nymphs

**Figure 1.**
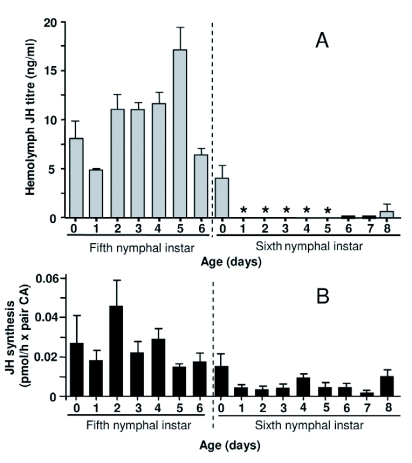
A. Juvenile hormone III (JH) titer in the hemolymph of fifth and sixth nymphal instar females of *Blattella germanica*. Values represent the mean ± SEM (*n* = 3–5). Asterisks mean that JH III was not detectable under our conditions of analysis. B. Rates of JH III synthesis by *corpora allata* (CA) incubated *in vitro* from fifth and sixth nymphal instars of *B. germanica*, according to data previously published (Cruz *et al.* 2003)

The concentration of JH III, which was the only JH homolog present in all samples, was determined in the hemolymph of *B. germanica* females during the two last nymphal instars (Fig. 1A). The titer of JH III in the hemolymph of the fifth (penultimate) nymphal instar was low, between 5 and 20 ng/ml. The most frequent values were around 10 ng/ml. Lower values were found on days 1 and 6, and higher values on day 5. Differences between values of day 5 and days 1 and 6 are statistically significant (*t*-test, p = 0.006 and p = 0.012, respectively).

Within the sixth (last) nymphal instar, maximal levels of JH III in the hemolymph were measured just after the molt (5 ng/ml). During the following 1 to 7 days JH III was undetectable or found at very low concentration, and increased significantly on day 8, reaching *ca*. 2 ng/ml.

According to our previous results (Cruz et al. 2003), values of JH III biosynthesis by CA during the penultimate nymphal instar are remarkably low (0.06 pmol/hour per CA pair, on day 2) and virtually constant, given that differences between the different days are not statistically significant, whereas those of the last nymphal instar are even lower (around 0.01 pmol/hour per CA pair when rates are maximal) (Fig. 1B). The JH synthesis values of Cruz et al. (2003) have been routinely confirmed in our laboratory. These values are in broad correspondence with hemolymph titer values when comparing the corresponding instars. However, the low and virtually constant synthesis values measured in fifth instar nymphs contrast with the moderately high and peaking titers of the same instar. Conversely, in the sixth instar nymphs, the low, but still measurable synthesis values correspond to practically null titers during most of the instar.

### JH in the adult female

**Figure. 2:**
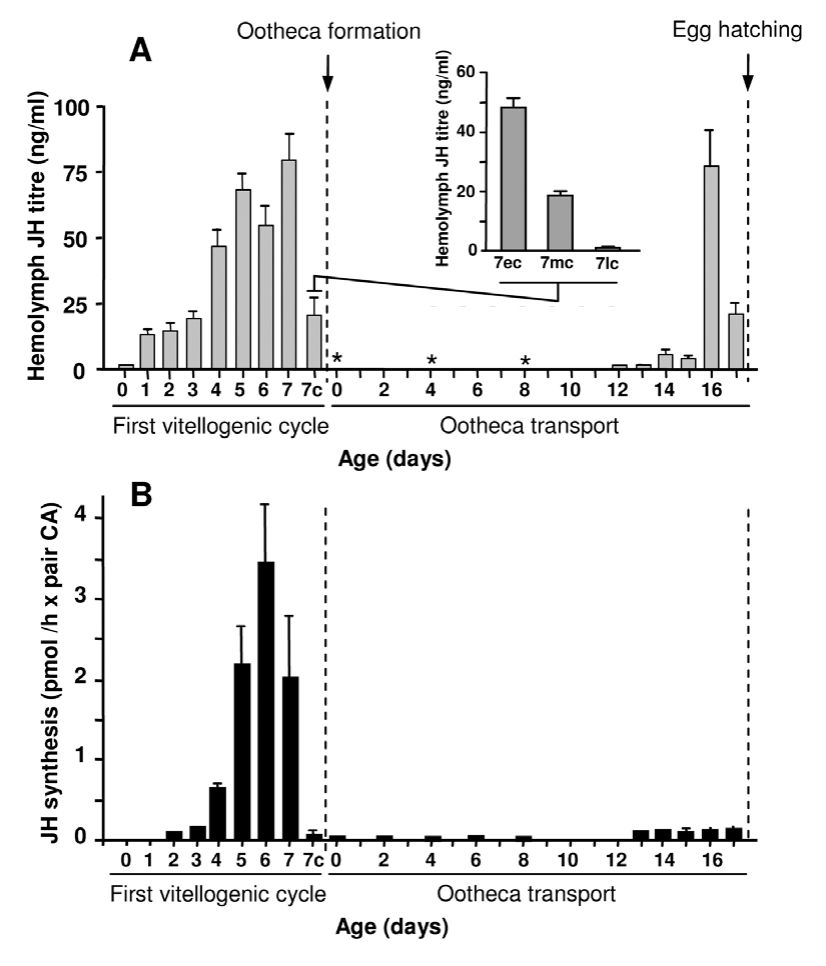
A. Juvenile hormone III (JH) titer in the hemolymph of adult females of *Blattella germanica*, during the first vitellogenic cycle and during the period of ootheca transport. Values represent the mean ± SEM (*n* = 3–5). The 7c means 7-day-old females with basal oocytes during chorion formation. JH titer in females that are forming the chorion are further detailed in the inset, showing the values during early chorion formation (7ec), middle chorion formation (7mc) and late chorion formation (7lc). Asterisks mean that JH III was not detectable under our conditions of analysis. B. Rates of JH III synthesis by *corpora allata* (CA) incubated *in vitro* from females of *B. germanica* in the first vitellogenic cycle, according to data previously published (Maestro *et al* 1994; Cruz *et al* 2003) (n = 7–15), and during the period of ootheca transport (n = 3–5).

JH III titer was determined in the hemolymph of adult females during the first vitellogenic cycle, and while transporting the ootheca (Fig. 2A). Just after the imaginai molt, JH III titer was similar to that measured in the last day of sixth nymphal instar. Then it increased steadily until about 70 ng/ml was present in 5 day-old females. The titer decreased on day 6, and increased again until it reached about 80 ng/ml in 7 day-old females, when basal oocytes began choriogenesis. Thereafter, JH III titer decreased dramatically to practically undetectable levels, which coincided with the last hours of choriogenesis (Fig. 2A, inset).

The pattern of JH III synthesis by the CA during the first vitellogenic cycle (Fig. 2B) was reported by Maestro et al. 1994 and Cruz et al. 2003. These results have been routinely confirmed in our laboratory. The pattern of synthesis is similar to that of JH III titer in the hemolymph (Fig. 2A), with the exception of a disparity on day 6, where JH synthesis is higher than on days 5 or 7 whereas JH titer is lower on day 6 relative to days 5 and 7.

After the first vitellogenic cycle, hemolymph JH III remains undetectable during most of the period of ootheca transport (Fig. 2A), but becomes detectable again from day 12 to day 15 (from ca. 1 ng/ml to ca. 5 ng/ml), increases dramatically on day 16 (ca. 75 ng/ml), and decreases on day 17 (ca. 20 ng/ml) which is when the ootheca is deposited (Fig. 2A). JH III titer during the first day of the second vitellogenic cycle is similar to that of the last day of ootheca transport (not shown).

JH III synthesis by the CA in females transporting the ootheca is very low during the first two thirds of this period, and only increases slightly in the last 5 days, previous to ootheca drop and egg hatching (Fig. 2B). The pattern during these last 5 days (Fig. 2B) contrasts with that of JH III titer in the hemolymph (Fig. 2A), the most important disparity appearing on day 16, when JH synthesis is low and JH titer is very high.

## Discussion

Combined gas chromatography and selected ion monitoring mass spectrometry showed that only JH III is detected in the hemolymph of female nymphs and adults of *B. germanica* confirming the previous results of Camps et al. (1987) and Sevala et al. 1999. In addition, JH III was the only homologue found in other species of cockroaches, such as *Leucophaea maderae, Nauphoeta cinerea, Blatta orientalis, Diploptera punctata* and *Periplaneta americana* (see Baker et al. 1984, and references therein).

In the penultimate nymphal instar, both JH III titer in the hemolymph and JH III production by the CA are measurable. The contrast between the low and constant synthesis values and the moderately high and peaking concentration values suggests that mechanisms of JH degradation are attenuated. In the last instar, however, JH titer becomes very low or undetectable, although JH synthesis is detected in certain days and can even approach the levels observed in penultimate instar. The virtual absence of JH in the hemolymph in the last nymphal instar has been observed in other cockroaches, such as *D. punctata* (Szibbo et al. 1982; Tobe et al. 1985) and *N. cinerea* (Lanzrein et al. 1975), and is important during the metamorphic molt that leads to the adult stage. In *D. punctata*, JH titer in the hemolymph is inversely correlated with levels of JH esterase activity (Szibbo et al. 1982). This may explain the fast clearing of hemolymph JH in the last nymphal instar (Fig. 1), and the lack of correspondence between JH titer and JH synthesis.

Maximal concentrations of JH III in the hemolymph occurred during the vitellogenic cycle in adult females, reaching a peak of *ca.* 80 ng/ml on day 7 (Fig. 2A). Our present values are somewhat higher than those preliminary reported (Camps et al. 1987), and fall within the same order of magnitude of those published by Sevala and co-workers (1999). In other cockroaches, maximal levels of circulating JH are also found in the adult vitellogenic female, for example, in *L. maderae*, in which up to 400 ng/ml have been measured (Engelmann and Mala 2000), and *D. punctata*, which shows the highest JH levels ever measured in a cockroach, reaching 1500 ng/ml in full vitellogenesis (Tobe et al. 1985).

In *B. germanica* JH induces vitellogenin synthesis (Martín et al. 1995; Comas et al. 1999; Comas et al. 2001). Increasing JH levels in the hemolymph of 3 to 5 day-old females correlate with the process of vitellogenesis and oocyte maturation. Interestingly, JH continues to increase until day 7 (Fig. 2), whereas vitellogenin production begins to decrease after day 5 (Martín et al. 1995). This suggests that JH has other functions, such as modulating vitellogenin incorporation into the oocyte (Martín et al. 1995). Indeed, the degree of enlargement of the intercellular spaces of the follicular epithelium correlates with the rates of JH synthesis and with the dynamics of basal oocyte growth (Pascual et al. 1992).

During most of the period of ootheca transport, *B. germanica* practically does not produce JH, which decreases oocyte growth and avoids the premature deposition of the ootheca (Tobe and Stay 1985; Osorio et al. 1997). Therefore, it is not surprising that hemolymph levels of JH III are very low in adult females during the first days of the period of ootheca transport, and that this correlates with the low levels of JH III synthesis measured in the CA (Fig. 2, see also Sevala et al. 1999). Interestingly, JH III synthesis and JH III titer begin to rise during the last 5 days of the transport period, as a prelude to the second vitellogenic cycle. However, whereas rates of JH III synthesis maintain moderate to low quite constant levels, hemolymph concentration shows a contrasting peak of *ca*. 75 ng/ml on the penultimate day (Fig. 2), which could be involved in the preparation of the ovaries for a new gonadotrophic cycle.

**Figure 3.**
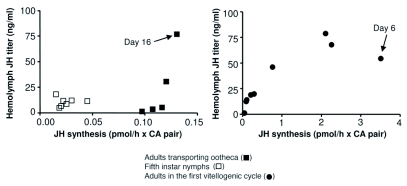
Relationship between juvenile hormone III (JH) synthesis by *corpora allata* (CA) incubated *in vitro*, and JH III titer in the hemolymph, in females of *Blattella germanica* in three stages: fifth instar nymphs, adults in the first vitellogenic cycle and adults transporting the ootheca. Data plotted are from figures 1 and 2.

In general, there is a significant positive correlation between JH synthesis and JH titer in females in penultimate and last instar nymphs, in adults during the first vitellogenic cycle, and in adults transporting the ootheca (Fig. 3). The main disparities appear on the penultimate day (day 6) of the first vitellogenic cycle of the adult female, when an increase in synthesis corresponds to a decrease in concentration, and on the penultimate day (day 16) of the period of ootheca transport, when high titer contrasts with low rates of synthesis (Fig. 3). These two stages are important in the reproductive cycle, the first one because of the shift from vitellogenesis to postvitellogenesis, and the second because of the reverse process. These disparities between synthesis and titer can be explained by a differential action of JH degrading enzymes. As stated above, hemolymph JH concentration has been shown to be inversely correlated with levels of JH esterase activity in the cockroach *D. punctata* (Szibbo et al. 1982). Unfortunately, JH esterases have yet to be measured over these stages in *B. germanica*. What is clear is that both parameters, JH synthesis and JH titer, are important in order to have a complete picture of the equilibrium between synthesis, degradation and biological effects of the hormone.
